# Supplementary material for: Heat adaptation of phage T7 under an extended genetic code
Source: Virus Evol. 2021 Dec 1;7(2):veab100. doi: 10.1093/ve/veab100 (PMC8923235; doi:10.1093/ve/veab100)
Supplement: veab100_Supp [file veab100_supp.zip › Sequencing_Analysis.zip!/Sequencing_Analysis/A1/evidence/RA_23.html]

BRESEQ :: Evidence


| Read alignment evidence... | | | | | | | | | | | |
| --- | --- | --- | --- | --- | --- | --- | --- | --- | --- | --- | --- |
|  | seq id | position | | ref | new | freq | score (cons/poly) | reads | annotation | genes | product |
| \* | NC\_001604.1 | 20,976 | 0 | A | C | 20.8% | 2835.8 / inf | 2566 | intergenic (–/–) | *–/–* | –/– |
| Reads supporting (aligned to +/- strand):  **ref** base A (992/1033);  **new** base C (276/256);  **total** (1273/1293) | | | | | | | | | | | |
| Fisher's exact test for biased strand distribution *p*-value = 2.42e-01 | | | | | | | | | | | |
| Kolmogorov-Smirnov test that lower quality scores support variant *p*-value = 9.80e-01 | | | | | | | | | | | |

Only 100 of 2594 total aligned reads displayed.  

`ACCTACCGGAACCGGAAGGGTCAAACTATAATCCCATGAAGCTGTACCGATTGTCTTCTTATGTGGTCCAACGAGACGCATTCGGCAACGTTCTGCAAATGGTGACTCGTGACCAGATAGCTTTTGGTGCTCTCCC‑TGAGGACATCCGTAAGGCTGTAGAAGGTCAAGGTGGTGAGAAGAAAGCTGATGAGACAATCGACGTGTACACTCACATCT‑ATCTGGATGAGGACTCAGGTGAATACCT‑CCGATACGAAGAGGTCGAGGGTATGGAAGTCCAAGGCTCCGATGGGACTTATCCTAAAGAGGCTTGCCCATACATCCCGATTCGGATGGTCAGACTAGATGGTGAATCCTACGGTCGTTCGTACATTGAGGAATACTTAGGTGACTTACGGTCCCTTGAAAATCTCCAAGAGGCTATCGTCAAGATGTCCATGATTAGCTCTAAGGTTATCGGCTTAGTGAATCCTGCTGGTATCACCCAGCCACGCCGACTGACCAAAGCTCAGACTGGTGACTTCGTTACTGGTCGTCCAGAAGACATCTCGTTCCTCCAACTGGAGAAGCAAGCAGACTTTACTGTAGCTAAAGCCGTAAGTG  >  NC_001604.1/20676‑21275`  

                                                                                                                                                                                                                                                                                                               `|`                                                                                                                                                                                                                                                                                                             
  `tgtgtatacgagacaGGTTCAAACCATAAGCCCATGAATCGTTACTGATTGTCTTCTTATGTGGTCCAACGAGACGCATTCGGCAATGTTCTGCAAATGGTGACTCGTGACCAGATAGCTTTTGGTGCTCTCCC‑TGAGGACACCCGTAAGGCTGTAGAAGGTCAAGGTGGTGAGAAGAAAGCTGATGAGACAATCGACGTGTACACTCACATCT‑ATCTGGATGAGGACTCAGGTAAATACCT‑CCGATACGAAGAGGTCGAGGGTATGGAAGTCCAAGGCTCCGATGTGACTTATCCTAAag                                                                                                                                                                                                                                                                                                           <  1:128112/287‑1 (MQ=255)`  
  `tcggagatgtgtataagagacagcTATAATCCCATGAAGATGTACCGATTGTCTTCTTATGTGGTCCAACGAGACGCATTCGGCAACGTTCTGCAAATGGTGACTCGTGACCAGATAGCTTTTGGTGCTCTCCC‑TGAGGACATCCGTAAGGCTGTAGAAGGTCAAGGTGGTGAGAAGAAAGCTGATGAGACAATCGACGTGTACACTCACATCT‑ATCTGGATGAGGACTCAGGTGAATACCT‑CCGATACGAAGAGGTCGAGGGTATGGAAGTCCAAGGCTCCGATGGGACTTATCCTAAag                                                                                                                                                                                                                                                                                                           <  1:436195/278‑1 (MQ=255)`  
  `gtaccGGAACCGGAAGGGTCAAACTATAATCCCATGAAGCTGTACCGATTGTCTTCTTATGTGGTCCAACGAGACGCATTCGGCAACGTTCTGCAAATGGTGACTCGTGACCAGATAGCTTTTGGTGCTCTCCC‑TGAGGACATCCGTAAGGCTGTAGAAGGTCAAGGTGGTGAGAAGAAAGCTGATGAGACAATCGACGTGTACACTCACATCT‑ATCTGGATGAGGACTCAGGTGAATACCT‑CCGATACGAAGAGGTCGAGGGTATGGAAGTCCAAGGCTCCGATGGGACTTATCCTAAag                                                                                                                                                                                                                                                                                                           <  1:116671/300‑1 (MQ=255)`  
   `taccGGAACCGGAAGGGTCAAACTATAATCCCATGAAGCTGTACCGATTGTCTTCTTATGTGGTCCAACGAGACGCATTCGGCAACGTTCTGCAAATGGTGACTCGTGACCAGATCGCTTTTGGTGATCTCCC‑TGAGGACATCCGTAAGGCTGTAGAAGGTCAAGGCGGTGTGAAGAAAGCTGATGAGACAATCGACGTGTACACTCACATCT‑ATCTGGATGATGACTCAGGTGAATACCT‑CCGATACGAAGAGGTCGAGGGTATGGAGGTCCAAGGCTCCGATGGGCCTTATCCTaacgc                                                                                                                                                                                                                                                                                                          >  1:428634/1‑298 (MQ=255)`  
        `gaaccggaaTGGTCAAACTATAATCCCATGAAGCTGTACCGATTGTCTTCTTATGTGGTCCAACGAGACGCATTCCGCAACGTTCTGCAAATGGTGACTCGTGACCAGATAGCTTTTTGTGCTCTCGC‑TGAGGACATCCGTTAGGCTGTAGACGGTCAAGGTGGTGAGAAGAAAGCTGATGAGACAATCTACGTGTACACTCAAATCT‑ATATGGATGAGGACTCAGGTGATTACCA‑CCGATCCGAAGAGGACGAGAGTATGGAAGTCCAAGGCGCCGATTGGACGTAGCCTAAAGTCGCtt                                                                                                                                                                                                                                                                                                     >  1:54911/1‑301 (MQ=255)`  
        `gaaccggaaGGGTCAAACTATAATCCCATGAAGCTGTACCGATTGTCTTCTTATGTGGTCCAACGAGACGCATTCGGCAACGTTCTGCAAATGGTGACTCGTGACCAGATAGCTTTTGGTGCTCTCCC‑TGAGGACATCCGTAAGGCTGTAGAAGGTCAAGGTGGTGAGAAGAAAGCTTATGAGACAGTCGACGTGTACACTCACATAT‑GTCTGGATGAGAACTCAGGTAAATACCT‑CCGATACGAAGAGGTCGAGGGTATGGAGGTCCAAGGCTCCGATGGGATTTATCCTACAGAGACtt                                                                                                                                                                                                                                                                                                     >  1:292613/1‑301 (MQ=255)`  
            `ggagatgtgtataagagacagccATGAAGCTGTACCGATTGTCTTCTTATGTGGTACAACGAGACGCATTCGGCAACGTTCTGCAAATGGTGACTCGTGACCAGATAGCTTTTGGTGCTCTCCC‑TGAGGACATCCGTAAGGCTGTAGAAGGTCAAGGTGGTGAGAAGAAAGCTGATGAGACAATCGACGTGTACACTCACATCT‑ATCTGGATGAGGACTCAGGTGAATACCT‑CCGATACGAAGAGGTCGAGGGTATGGAAGTCCAAGGCTCCGATGGGACTTATCCTAAAGAGGCTTGccc                                                                                                                                                                                                                                                                                                 <  1:120687/280‑1 (MQ=255)`  
                `aGGGTCAAACAATAGTCCCATGAAGCTGTACCGATTGTCTTCTTATGTGGTCAAACGAGACGCATTCGGCAACGTTATGCAAATGGTGACTCGTGACCAGATAGCTTTTGGTGCTCTCCC‑TGAGGACATCCGTAAGGCTGTAGAAGGTCAAGGTGGTGAGAAGAAAGCTGATGAGACAATCGACGTGTACACTCACATCT‑ATCTGGATGAGGACTCAGGTGAATACCT‑CCGATACGAAGAGGTCGAGGGTATGGAAGTCCAAGGCTCCGATGGGACTTATCCTAAAGAGGCTTGCCCATAc                                                                                                                                                                                                                                                                                             <  1:59303/301‑1 (MQ=255)`  
                 `gggTCAAACTATAATCGCATGATGCAGTACCGATTTTCTTCTTATGTGGTCCAAAGTGACGCATTCGGCAACGTTCTGCAAATGGTGACTCGTTACCAGATAGCTTTTGGTGCTCTCCC‑TGAGGACATCCGTAAGGCTGTAGAAGGTCAAGGTGGTGAGAAGAAAGCTGATTATACAATCGACGTGTACCCTCACATCT‑ATCTGGATGATGACTCAGGTAAATACCT‑CCGATACGAAGAGGTCGAGGGTATGGAAGTCCAAGGCTCCGTTGGGACTTATCCTAAAGAGGCTTGCCCATACa                                                                                                                                                                                                                                                                                            <  1:353499/301‑1 (MQ=255)`  
                   `gTCAAACTATAATCCCATGAAGCTGTACCGATTGTCTTCTTATGTGGTCCAACGAGACGCATTCGGCAACGTTCTGCAAATGGTGACTCGTGACCAGATAGCTTTTGGTGCTCTCCC‑TGAGGACATCCGTAAGGCTGTAGAAGGTCAAGGTGGTGAGAAGAAAGCTGATGAGACAATCGACGTGTACACTCACATCT‑ATCTGGATGAGGACTCAGGTGAATACCT‑CCGATACGAAGAGGTCGAGGGTATGGAAGTCCAAGGCTCCGATGGGACTTATCCTACAGAGGCTTGCCCATACATc                                                                                                                                                                                                                                                                                          >  1:87100/1‑301 (MQ=255)`  
                    `tCAAACTATAATCCCATGAAGCTGTACCGATTGTCTTCTTATGTGGTCCAACGAGACGCATTCGGCAACGTTCTGCAAATGGTGACTCGTGACCAGATAGCTTTTGGTGCTCTCCC‑TGAGGACAACCGTAAAGCTGTAGAAGGTCAAGGTGGTGAGAAGAAAGCTGATGAGACAATCGACGTGTACACTCACATCT‑ATCTGGATGAGGACTCAGGTGAATACCT‑CCGATACGAAGAGGTCGAGGGTATGGAAGTCCAAGGCTCCGATGGGACTTATCCTAAAGAGGCTTGCCCATACATcc                                                                                                                                                                                                                                                                                         <  1:467959/301‑1 (MQ=255)`  
                                  `cATGAAGCTGTACCGATTGTCTTCTTATGTGGTCCAACGAGACGCATTCGGCAACGTTCTGCAAATGGTGACTCGTGACCAGATAGCTTTTGGTGCTCTCCC‑TGAGGACATCCGTAAGGCTGTAGAAGGTCAAGGTGGTGAGAAGAAAGCTGATGAGACAATCGACGTGTACACTCACATCT‑ATCTGGATGAGGACTCAGGTGAATACCT‑CCGATACGAAGAGGTCGAGGGTATGGAAGTCCAAGGCTCCGATGGGACTTATCCTACAGAGGCTTGCCCATACATCCCGATTCGGATGGtt                                                                                                                                                                                                                                                                           >  1:403410/1‑300 (MQ=255)`  
                                    `tGAAGCTGTACCGATTGTCGTCTTATGTGGTCCAACGAGACGCATTCGGAAACGTTCTGCAAATGGTGACTCGTGACCAGATAGCTTTTGGTGCTCTCCC‑TGAGGACATCCGTAAGGCTGTAGAAGGTCAAGGTGGTGAGAAGAAAGCTGATGAGACAATCGACGTGTACACTCACATCT‑ATCTGGATGAGGACTCAGGTGAATACCT‑CCGATACGAAGAGGTCGAGGGTATGGAAGTCCAAGGCTCCGATGGGACTTATCCTAAAGAGGCTTGCCCATACATCCCGATTCGGATGGTCAg                                                                                                                                                                                                                                                                         <  1:145327/301‑1 (MQ=255)`  
                                      `aaGCTGTACCGATTGTCTTCTTATGTGGTCCAACTAGACGCATTCGGCAACGTTCTGCAAATGGTGACTCGTGACCAGATAGCTTTTGGTGCTCTCCC‑TGAGGACATCCGTAAGGCTGTAGAAGGTCAAGGTGGTGAGAAGAAAGCTGATGAGACAATCGACGTGTACACTCACATCT‑ATCTGGATGAGGACTCAGGTGAATACCT‑CCGATACGAAGAGGTCGAGGGTATGGAAGTCCAAGGCTCCGATGGGCCTTATCCTAAAGATGCTTGACCGTACATCCCGATTCGGATGGTCAGaa                                                                                                                                                                                                                                                                       >  1:331224/1‑300 (MQ=255)`  
                                      `aaGCTGTACCGATTGTCTTCTTATGTGGTCCAACGAGTCGCATTCGGCAACGTTCTGCAAATGGTGACTCGTGACCAGATAGCTTTTGGTGCTCTCCC‑TGAGGACATCCGTAAGGCTGTAGAAGGTCAAGGTGGTGAGAAGAAAGCTGATGAGACAATCGACGTGTACAATCACATCT‑ATCTGGATGAGGACTCAGGTGAATACCT‑CCGATAAGAAGAGGTCGAGGGTATGGAAGTCCAAGGCTCCGATGGGACGTATCCTAAAGAGGCTTGCCCATACATCCCGATTCGGATGGTCAGAc                                                                                                                                                                                                                                                                       >  1:77992/1‑301 (MQ=255)`  
                                           `gTACCGATTGTCTTCTTATGTGGTCCAACGAGACGCATTCGGCAACGTTCTGCAAATGGTGACTCGTGACCAGATAGCTTTTGGTGCTCTCCC‑TGAGGACATCCGTAAGGCTGTAGAAGGTCAAGGTGGTGAGAAGAAAGCTGATGAGACAATCGACGTGTACACTCACATCT‑ATCTGGATGAGGACTCAGGTGAATACCT‑CCGATACGAAGAGGTCGAGGGTATGGAAGTCCAAGGCTCCGATGGGACTTATCCTAAAGAGGCTTGCCAATACATCCCGATTCGGATGGTCAGACTAGAt                                                                                                                                                                                                                                                                  >  1:131435/1‑301 (MQ=255)`  
                                           `gTACCGATTGTCTTCTTATGTGGTCCAACGAGACGCATTCAGCAACGTTCTGCAAATGGTGACTCGTGACCAGATAGCTTTTGGTGCTCTCCC‑TGAGGACATCCGTAAGGCTGTAGAAGGTCAAGGTGGTGAGAAGAGAGCTGATGAGACAATCGACGTGTACACTCACATCT‑ATCTGGATGAGGACTCAGGTGAATACCT‑CCGATACGAAGAGGTCGAGGGTATGGAAGTCCAAGGCTCCGATGGGACTTATCCTACAGAGGCTTGCCAATACATCCCGATTCGGATGGTCAGAcctgtc                                                                                                                                                                                                                                                                  >  1:237564/1‑296 (MQ=255)`  
                                            `tACCTATTGTCTTCTTATGTGGTCCAACGAGACGCATTCGGCAACGTTCTGCAAATGGTGACTCGTGACCAGATAGCTTTTGGTGCTCTCCC‑TGAGGACATCCGTAAGGCTGTAGAAGGTCAAGGTGGTGAGAAGAAAGCTGATGAGACAATCGACGTGTACACTCACATCT‑ATCTGGATGAGGACTCAGGTGAATACCT‑CCGATACGAAGAGGTCGAGGGTATGGAAGTCCAAGGCTCCGATGGGACTTATCCTAAAGAGGCTTGCCCATACATCCCGATTCGGATGGTCAGACTAGATg                                                                                                                                                                                                                                                                 >  1:9507/1‑301 (MQ=255)`  
                                             `aCCGATTGTCTTCTTATGTGGTCCAACGAGACGCATTCGGCAACGTTCTGCAAATGGTGACTCGTGACCAGATAGCTTTTGGTGCTCTCCC‑TGAGGACATCCGTAAGGCTGTAGAAGGTCAAGGTGGTGAGAAGAAAACTGATGAGACAATCGACGTGTACACTCACATCT‑ATCTGGATGAGGACTCAGGTGAATACCT‑CCGATACGAAGAGGTCGAGGGTATGGAAGTCCAAGGCTCCGATGGGACTTATCCTAAAGAGGCTTGCCCATACATCCCGATTCGGATGGTCAGACTAGATgg                                                                                                                                                                                                                                                                >  1:240997/1‑301 (MQ=255)`  
                                                    `agagacagaTGTGGTCCAACGAGACGCATTCGGCAACGTTCTGCAAATGGTGACTCGTGACCAGATAGCTTTTGGTGCTCTCCC‑TGAGGACATCCGTAAGGCTGTAGAAGGTCAAGGTGGTGAGAAGAAAGCTGATGAGACAATCGACGTGTACACTCACATCT‑ATCTGGATGAGGACTCAGGTGAATACCT‑CCGATACGAAGAGGTCGAGGGTATGGAAGTCCAAGGCTCCGATGGGACTTATCCTAAAGAGGATTGCCCATACATCCCGATTCGGATGGTCAGACTAGATGGTGAATcc                                                                                                                                                                                                                                                         <  1:197332/293‑1 (MQ=255)`  
                                                      `cttcttATGTGGTCCAACGAGACGCATTCGGCAACGTTCTGCAAATGGTGACTCGTGACCAGATAGCTTTTGGTGCTCTCCC‑TGAGGACATCCGTAAGGCTGTAGAAGGTCAAGGTGGTGAGAAGAAAGCTGATTAGACAATCGACGTGTACACTCACATCT‑ATCTGTATGAGGACTCAGGTGAATACCT‑CCGATACGAAGATGTCGAGGGTATGGAAGTCCAAGGCTCCGATGGGACTTATCCTAAAGAGGCTTGCCAATACATCCCGATTCGGATGGTCAGACTAGATGGTGAATCCTa                                                                                                                                                                                                                                                       >  1:109102/1‑301 (MQ=255)`  
                                                         `cttATGTGGTCCAACGAGACGCATTCGGCAACGTTCTGCAAATGGTGACTCGTGACCAGATAGCTTTTGGTGCTCTCCC‑TGCTGACATCCGTAAGGCTGTAGAAGGTCAAGGTGGTGATAAGAAAGCTGATGAGACAATCGACGTGTTCACTCACATCT‑ATCTGGATGAGGATTAAGGTGAATACCT‑CCGATACGAAGAGGTCGAGGGTATGGAAGACCAAGGCTCCGATGGGACTTATCCTAAAGAGGCTTGCCCATACATCCCGATCCGGAGGGCCAGCCTAGATGGTGAATccctgct                                                                                                                                                                                                                                                    >  1:167991/1‑296 (MQ=255)`  
                                                          `tgggtataagagacaggaCGCATTCGGCAACGTTCTGCAAATGGTGACTCGTGACCATATAGCTTTTGGTGCTCTCCC‑TGCGGACATCCGTAAGGCGGTAGAAGTTCAAGGTGGTGAGAAGAAAGCTGATGAGACAATCGACTTGTACACTCACATCT‑ATCTGGATGAGGACTCAGGTGAATACCT‑CCGATACGAAGAGGTCGAGGGTATGGAAGTCCAAGGCTCCGATGGGACTTATCCTAAAGAGGCTTGCCCATACATCCCTATTCGGATGGTCAGACTAGATGGTGAATCCTACGGt                                                                                                                                                                                                                                                   <  1:367611/285‑1 (MQ=255)`  
                                                                `agTCCAACGAGACGCATTCGGCAACGTTCTGCAAATGGTGACTCGTGACCAGATAGCTTTTGGTGCTCTCCC‑TGAGGACATCCGTAAGGCTGTAGAAGGTCAAGGTGGTGAGAAGAAAGCTGATGAGACAATCGACGTGTACACTCACATCT‑ATCTGGATGAGGACTCAGGTGAATACCT‑CCGATACGAAGAGGTCGAGGGTATGGAAGTCCAAGGCTCCGATGGTACTTATCCTAAAGAGGCTTGCCCATACATCCCGATTCGGATGGTCAGACTAGATGGTGAATCCTACGGtcgttcg                                                                                                                                                                                                                                             >  1:17666/2‑301 (MQ=255)`  
                                                                    `aaaCGGGCCGAATTCGGAAACGTTCGGCAAATGTGGCCTCGTGACCGGATAGCTTTTGGGGCTCTCCT‑TGAGGACATCCGTAAGGCGGAAGAAGTTAAAGGGGGTGAGAAGAAAGTGGATGAGACAACCGACGTGAACACT‑ACAACTCATTGGGATAAGGA‑TCAAGTGGAAACCTCCCGATACGAAGAGGTGGAGGGAATGGAAGCCAAAGGCCCGGATGGGACTAATCTTAAAGAGG‑TTGCCCATACATCCCGATTCGGATGGTCAGACTAGATGGTGAATCCTACGGTCGTTAGTACAt                                                                                                                                                                                                                                        <  1:190105/300‑1 (MQ=255)`  
                                                                     `aaCGAGACGCATTGGGAAACGGTCTGCAAATGGGGACTCGGGAGCAGATAGCTTTGGGTGCTCTCCC‑TGGGGACATCCGTAAGGCTGTAGAAGGTCAAGGTGGCGAGAAGAAAGCTGATTAGACAATCGACGTTTACACTCACATCT‑ATGTGGATGAGGACTCAGGTGAATACCT‑CCGATACGAAGAGGTCGAGGGTATGGAAGTCCAAGGCTCCGATGGGACTTATCCTAAATCGGCTTGCCCATACATCCCGATTCGGATGGTCAGACTAGATGGTGAATCCTACGGTCGTTCGTACAt                                                                                                                                                                                                                                        <  1:287807/301‑1 (MQ=255)`  
                                                                     `aaCGAGACGCATTCGGCAACGTTCTGCAAATGGTGACTCGTGACCAGATAGCTTTTGGTGCTCTCCC‑TGAGGACATCCGTAAGGCTGTAGAAGGTCAAGGTGGTGAGAAGAAAGCTGATGAGACAATCGACGTGTACACTCACATCT‑ATCTGGATGAGGACTCAGGTGAATACCT‑CCGATACGAAGAGGTCGAGGGTATGGAAGTCCAAGGCTCCGATGGGACTTATCCTAAAGAGGCTTGCCCATACATCCCGATTCGGATGGTCAGACTAGATGGTGAATCCTACGGTCGTTCGTACAt                                                                                                                                                                                                                                        <  1:136577/301‑1 (MQ=255)`  
                                                                     `aaCGAGACGCATTCGGCAACGTTCTGCAAATGGTGACTCGTGACCAGATAGCTTTTGGTGCTCTCCC‑TGAGGACATCCGTAAGGCTGTAGAAGGTCAAGGTGGTGAGAAGAAAGCTGATGAGACAATCGACGTGTACACTCACATCT‑ATCTGGATGAGGACTCAGGTGAATACCT‑CCGATACGAAGAGGTCGAGGGTATGGAAGTACAAGGCTCCGATGGGACTTATCCTAAAGAGGCTTGCCCATACATCCCGATTCGGATGGTCAGACTAGATGGTGAATCCTACGGTCGTTCGTACAt                                                                                                                                                                                                                                        <  1:502787/301‑1 (MQ=255)`  
                                                                     `aaCGAGACGCATTCGGCAACGTTCTGCAAATGGTGACTCGTGACCAGATAGCTTTTGGTGCTCTCCC‑TGAGGACATCCGTAAGGCTGTAGAAGGTCAAGGTGGTGAGAAGAAAGCTGATGAGACAATCGACGTGTACACTCACATAT‑ATCTGGATGAGGACTCAGGTGAATACCT‑CCGATACGAAGAGGTCGAGGGTATGGAAGTCCAAGGCTCCGATGGGACTTATCCTAAAGAGGCTTGCCCATACATCCCGATTCGGATGGTCAGACTAGATGGTGAATCCTACGGTCGTTCGTACAt                                                                                                                                                                                                                                        <  1:34740/301‑1 (MQ=255)`  
                                                                     `aaCGAGACGCATTCGGCAACGTTCTGCAAATGGGGACTCGTGACCAGATAGCTTTTGGTGCTCTCCC‑TGAGGACATCCGTAAGGCTGTAGAAGGTCAAGGTGGTGAGAAGAAAGCTGATGAGACAATCGACGTGTACACTCACATCT‑ATCTGGATGAGGACTCAGGTGAATACCT‑CCGATACGAAGAGGTCGAGGGTATGGAAGTCCAAGGCTCCGATGGGACTTATCCTAAAGAGGCTTGCCCATACATCCCGATTCGGATGGTCAGACTAGATGGTGAATCCTACGGTCGTTCGTACAt                                                                                                                                                                                                                                        <  1:446685/301‑1 (MQ=255)`  
                                                                        `gagaCGCATTCGGCCACGTTCTGCAAATGGTGACTCGTGACCAGATAGCTTTTGGTGCTCTCCC‑TGAGGACATTCGTAAGGCTGTAGAAGGTCAAGGTGGTGATAAGAAAGCTGATGAGACAATTGACGTGTACACTCACATCT‑ATCTGGATGAGGACTCAGGTGAATACCT‑CCGATACGAAGAGGTCGAGGGTATGGAAGTCCAAGGCTCCGATGGGACTTATCCTAAAGAGGCTTGCCCATACATCCCGATTCTGATGGTGAGACTAGATGGTGAATCCTCCGGTCGTTCGTACATTgt                                                                                                                                                                                                                                     >  1:509680/1‑300 (MQ=255)`  
                                                                         `agaCGCATTCGGCAACGTTCTGCAAATGGTGACTCGGGACCAGAGAGCTTTTGGTGCTCTCAC‑TGAGGACATCCGTAAGGCTGTAGAAGGTCAAGGTGGTGAGAAGAAAGCTGATGAGACAATCGACGTGTACACTTACATCG‑ATCTGGATGAGGACTCAGGTGAATACCT‑CCGATACGAAGAGGTCGAGGGTATGGAAGTCCAAGGCTCCGATGGGACTTATCCTAAAGAGGCTTGCCCATACATCCCGATTCGGATGGTCAGACTAGATGGTGAATCCTACGGTCGTTCGTACATTGAg                                                                                                                                                                                                                                    <  1:83520/301‑1 (MQ=255)`  
                                                                               `aTTCGGCAACGTTCTGCAAATGGTGACTCGTGACCAGATAGCTTTTGGTGCTCTCCC‑TGAGGACATCCGTAAGGCTGTAGAAGGTCAAGGTGGTGAGAAGAAAGCTGATGAGACAATCGACGTGTACACTCACATCT‑ATCTGGATGAGGACTCAGGTGAATACCT‑CCGATACGAAGAGGTCGAGGGTATGGAAGTCCAAGGCTCCGATGGGACTTATCCTAAAGAGGCTTACCCATACATCCCGATTCGGATGGTCAGACTAGATGGTGAATCCTACGGTCGTTCGTACATTGAGGAATAc                                                                                                                                                                                                                              <  1:149730/301‑1 (MQ=255)`  
                                                                                 `ccGGCAACGTTCTGCAAATGGTAACTCGTGACCAGATAGCTTTTGGTGCTCTCCC‑TGAGGACAGCCATAAGGGTGTAGAAGGTCGAGGTGGTGCGGAGAAAGCTGATGAGACAAACGACGAGTACACTCAAATTT‑ATCTGGATGAGGACTCAGGTTAATACCT‑CCGATACGAAGAGGTCGAGGGTATGGAAGTCCAAGGGTCCGATGGGCCTTTTCCTAAAGAGGCTTGCCCATACATCCCGATTCGGATGGTCAGACTAGATGGTGAATCCTACGGTCGTTCGTACATTGAGGAATACtt                                                                                                                                                                                                                            <  1:424309/300‑1 (MQ=255)`  
                                                                                  `gggCAACGTTCTGAAAATGGTGACTCGTGACCAGATAGCTTTTGGTGCTCTCCC‑TGAGGACATCCGTAAGGCTGTAGAAGGTCAAGGTGGTGAGAAGAAAGCTGATGAGACAATCGACGTGTACACTCACATCT‑ATCTGGATGAGGACTCAGGTGAATACCT‑CCGATACGAAGAGGTCGAGGGTATGGAAGTCCAAGGCTCCGATGGGACTTATCCTAAAGAGGCTTGCCCATACATCCCGATTCGGATGGTCAGACTAGATGGTGAATCCTACGGTCGTTCGTACATTGAGGAATACTTa                                                                                                                                                                                                                           <  1:471001/300‑1 (MQ=255)`  
                                                                                    `gCAACGTTCTGCAAATGGTGACTCGTGACCAGATAGCTTTTGGTGCTCTCCC‑TGAGGACATCCGTAAGGCTGTAGAAGGTCAAGGTGGTGAGAAGAAAGCTGATGAGACAATCGACGTGTACACTCACATCT‑ATCTGGATGAGGACTCAGGTGAATACCT‑CCGATACGAAGAGGTCGAGGGTATGGAAGTCCAAGGCTCCGATGGGACTTATCCTAAAGAGGCTTGCCCATACATCCCGATTCGGATGGTCAGACTAGATGGTGAATCCTACGGTCGTTCGTACATTGAGGAATACTTAgg                                                                                                                                                                                                                         <  1:306957/301‑1 (MQ=255)`  
                                                                                        `cGTTCTGCAAATGGTGTCTCGTGACCAGAGAGGTTTTGGTGCTCTCCC‑TGAGGAAATACGTAAGGCTGGAGAAGGTAAAGGTGGTGAGAAGAAAGCTGATGAGACAATCGACGTGTACACTCAAAGCT‑ATCTGGATGAGGAATCAGGTGAATACCT‑CCGATACGAAGAGGTCGAGGGTATGGAAGTCCAAGGCTCCGATGGGACTTATCCTAAAGAGGCTGGCCCATACATCCAGATTCGGATGGTCAGACTAGATGGTGAATCCTACGGTCGTTCGTACATTGAGGAATACTTAGGTGAc                                                                                                                                                                                                                     <  1:483159/301‑1 (MQ=255)`  
                                                                                           `tCTGCAAATGGTGACTCGTGACCAGATAGCTTTTGGTGCTCTCCC‑TGAGGACATCCGTAAGGCTGTAGAAGGTCAAGGTGGTGAGAAGAAAGCTGATGAGACAATCGACGTGTACACTCACATCT‑ATCTGGATGAGGACTCAGGTGAATACCT‑CCGATACGAAGAAGTCGAGGGTATGGAAGTCCAAGGCTCCGATGGGACTTATCCTACAGAGGCTTGCCCATCAATCCCGATTCGGATGGTCAGACTAGATGGTGAATCCTCCGGTCGTCCGTACATTGAGGAATACTTAGGTGACtga                                                                                                                                                                                                                  >  1:297122/1‑299 (MQ=255)`  
                                                                                           `aaggCAAGTAGTGACTTGTGACAAGATAGCTGTCTGTGCTATCCCGTGTGTA‑ATTCGTAAGGCTTTAGAAGGTGACGGTGGTGAGAAGAAATCTTATTAGATGTTTGACTTGTACACTTACATCT‑ATCTGGATGATTACTCAGGTGAATACCT‑CCGATACGAAGAGGTCGAGGGTATGGAAGTCCAAGGCTCCGAGTGTACTTATCCGACAGAGGGTTGTCCATACATCCCGATTTGGATGGGCAGACTAGATGGTGAATCCTACGGTCGTTCGTACATTGAGGAATACTTAGGTGACTTa                                                                                                                                                                                                                  <  1:260273/298‑1 (MQ=255)`  
                                                                                            `cTGCAAATGGTGACTCGTGACCAGATAGCTTTTGGTGCTCTCCC‑TGAGGACATCCGTAAGGCTGTAGAAGGTCAAGGTGGTGAGAAGAAAGCTGATGAGACAATCGACGTGTACACTCACATCT‑ATCTGGATGAGGACTCAGGTGAATACCT‑CCGATACGAAGAGGTCGAGGGTATGGAAGTCCAAGGCTCCGATGGGACTTATCCTAAAGAGGCTTGCCCATACATCCCGATTCGGCTGGTCAGACTAGATGGTGAATCCTACGGTCGTTCGTACATTGAGGAATACTTAGGTGACTTAc                                                                                                                                                                                                                 >  1:441495/1‑301 (MQ=255)`  
                                                                                            `cTGCAAATGGTGACTCGTGACCAGATAGCTTTTGGTGCTCTCCC‑TGAGGACATCCGTAAGGCTGTAGAAGGTCAAGGTGGTGAGAAGAAAGCTGATGAGACAATCGACGTGTACACTCACATCT‑ATCTGGATGAGGACTCAGGTGAATACCT‑CCGATACGAAGAGGTCGAGGGTATGGAAGTCCAAGGCTCCGATGGGACTTATCCTAAAGAGGCTTGCCCATACATCCCGATTCGGATGGTCAGACTAGATGGTGAATCCTACGGTCGTTCGTACAtctgtctcttatacacatctccga                                                                                                                                                                                                                 >  1:199849/1‑278 (MQ=255)`  
                                                                                              `gCAAATGGTGACTCGTGACCAGATAGCTTTTGGTGCTCTCCC‑TGAGGACATCCGTAAGGCTGTAGAAGGTCAAGGTGGTGAGAAGAAAGCTGATGAGACAATCGACGTGTACACTCACATCT‑ATCTGGATGAGGACTCAGGTGAATACCT‑CCGATACGAAGAGGTCGAGGGTATGGAAGTCCAAGGCTCCGATGGGACTTATCCTACAGAGGCTTGCCCATACATCCCGATTCGGATGGTCAGACTAGATGGTGAATCCTACGGTCGTTCGTACATTGAGGAATACTTAGGTGACTTACgg                                                                                                                                                                                                               <  1:1411/301‑1 (MQ=255)`  
                                                                                                 `cggagatgtgtataagagacaggCTTTTGGTGCTCTCCC‑TGAGGACATCCGTAAGGCTGTAGAAGGTCAAGGTGGTGAGAAGAAAGCTGATGAGACAATCGACGTGTACACTCACATCT‑ATCTGGATGAGGACTCAGGTTAATACCT‑CCGAGACGAAGAGGTCGAGGGTATGGAAGTCCAAGGCTCCGATGGGACTTATCCTACAGAGGCTTGCCCATACATCCCGATTCGGATGGTCAGACTAGATGGTGAATCCTACGGTCGTTCGTACATTGAGGAATACTTAGGTGACTTACGGTcc                                                                                                                                                                                                            <  1:372944/279‑1 (MQ=255)`  
                                                                                                         `cTCGTGACCAGATAGCTTTTGGTGCTCTCCC‑TGAGGACATCCGTAAGGCTGTAGAAGGTCAAGGTGGTGAGAAGAAAGCTGATGAGACAATCGACGTGTACACTCACATCT‑ATCTGGATGAGGCCTCAGGTGAATACCT‑CCGATACGAAGAGGTCGAGGGTATGGAAGTCCAAGGCTCCGATGAGACTTATCCTACAGAGGCTTGCCCATACATCCCGATACGGAGGGTCAGACTAGATGGTGTATCCTACGGTAGTTCGTACATTGAGGAATACTTAGTTGACTTAAGGTCCCTTGcaaa                                                                                                                                                                                                    >  1:339413/1‑301 (MQ=255)`  
                                                                                                         `cTCGTGACCAGATAGCTTTTGGTGCTCTCCC‑TGAGGACATCCGTAAGGCTGTAGAAGGTCAAGGTGGTGAGAAGAAAGCTGATGAGACAATCGACGTGTACACTCACATCT‑ATCTGGATGAGGACTCAGGTGAATACCT‑CCGCTACTAAGAGGTCGAGGGTATGGAAGTCCAAGGCTCCGATGGGACTGATCCTAAAGAGGCTTGCCCATACATCCCGATCCGGATGGTCAGACTAGCTGGTGAATCCTACGGTCGTTTGTACATTTAGGAATACTTAGGTCACTTACGGTCCCtgtctct                                                                                                                                                                                                    >  1:392459/1‑295 (MQ=255)`  
                                                                                                             `tGACCAGATAGCTTTTGGTGCTCTCCC‑TGAGGACATCCGTAAGGCTGTAGAAGGTCAAGGTGGTGAGAAGAAAGCGGATGAGACAATCGACGTGTACACTCACATCT‑ATCTGGATGAGGACTCAGGTGAATACCT‑CCGATACGAAGAGGTCGAGGGTATGGAAGTCCAAGGCTCCGATGGGACTTATCCTAAAGAGGCTTGCCCATACATCCCGATTCGGATGGTCAGACTAGATGGTGAATCCTACGGTCGTTCGTACATTGAGGAATACTTAGGTGACTTACGGTCCCTTGAACAtctc                                                                                                                                                                                                >  1:398517/1‑301 (MQ=255)`  
                                                                                                                       `gCTTTTGGTGCTCTCCC‑TGAGGACATCCGTAAGGCTGTAGAAGGTCAAGGTGGTGAGAAGAAAGCTGATGAGACAATCGACGTGTACACTCACATCT‑ATCTGGATGAGGACACAGGTGAATACCT‑CCGATACGAAGAGGTCGAGGGTATGGAAGTCCACGGCTCCGATGGGACTTATCCTAAAGAGGCTTGCCCATACATCCCGATTCGGATGGTCAGACTAGATGGTGAATCCTACGGTAGTTCGTACATTGAGGACTACTTAGGTGACTCACGGTCCGTAGAAACTCTCCATGAGGcaa                                                                                                                                                                                      >  1:245390/1‑299 (MQ=255)`  
                                                                                                                        `cTTTTGGTGCTCTCCC‑TGAGGACATCCGTAAGGCTGTAGAAGGTCAAGGTGGTGAGAAGAAAGCTGATGAGACAATCGACGTGTACACTCCCATCT‑ATCTGGATGAGGACTCAGGTGAATACCT‑CCGATACGAAGAGGTCGAGGGTATGGAAGTCCAAGGCTCCGATGGGACTTATCCTAAAGAGGCTTGCCCATACATCCCGATTCGGATGGTCAGACTAGATGGTGAATCCTACTGTCGTTCGTACATTGAGGAATACTTAGGTGACTTACGGTCCCTTGAAAATCTCCAAGAGGCTAt                                                                                                                                                                                     >  1:421044/1‑301 (MQ=255)`  
                                                                                                                                `gcactacc‑TGAGGACATCCGGAAGGCTGTAGAAGGACAAGGTGGTGAGAAGAAAGCTGATGAGACAATAGACGTGTACACTCACATCT‑ATCTGGATGAGGACTCAGGTGAAGACCT‑CCGAGACGAAGAGGTCGAGGGTATGGAAGTCCAAGGCACCGATGGGACTTATCCTAAAGAGGCTTGACCATACAGCTCGATTCGGATGGTGAGAATAGATGGTGAATCCTACGGTTGTGAGTACATTGAGGAATACTTAGGTGACTTACGGTCCCTTGAAAATCTCCAAGAGGCTATCGTCAAGa                                                                                                                                                                             <  1:160162/295‑1 (MQ=255)`  
                                                                                                                                `gCTCTCCC‑TGAGGACATCCGTAAGGCTGTAGAAGGTCAAGGTGGTGAGAAGAAAGCTGATGAGACAATCGACGTGTACACTCACATCT‑ATCTGGATGAGGACTCAGGTGAATACCT‑CCGATACGAAGAGGTCGAGGGTATGGAAGTCCAAGGCTCCGATGGGACTTATCCTAAAGAGGCTTGCCCATACATCCCGATTCGGATGGTCAGACTAGATGGTGAATCCTACGGTCGTTCGTACATTGAGGAATACTTAGGTGACTTACGGTCCCTTGAAAATCTCCAAGAGGCTATCGTCAAGa                                                                                                                                                                             <  1:478975/301‑1 (MQ=255)`  
                                                                                                                                   `ctcCC‑TGAGGACATCCGTAAGGCTGTAGAAGGTCAAGGTGGTGAGAAGAAAGCTGATGAGACAATCGACGTGTACACTCACATCT‑ATCTGGATGAGGACTCAGGTGAATACCT‑CCGATACGAAGAGGTCGAGGGTATGGAAGTCCAAGGCTCCGATGGGACTTATCCTAAAGCGGCTTGCCCATACATCCCGATTCGGATGGTCAGACTAGATGGTGAATCCTACGGTCGTTCGTACATTGAGGAATACTTAGGTGACTTACGGTCCCTTGAAAATCTCCAAGAGGCTATCGTCAAGactg                                                                                                                                                                          >  1:223477/1‑298 (MQ=255)`  
                                                                                                                                     `ccc‑TGAGGACATCCGTAAGGCTGTAGAAGGTCAAGGTGGTGAGAAGAAAGCTGATGAGACAATCGACGTGTACACTCACATCT‑ATCTGGATGAGGACTCAGGTGAATACCT‑CCGATACGAAGAGGTCGAGGGTATGGAAGTCCAAGGCTCCGATGGGACTTATCCTAAAGAGGCTTGCCCATACATCCCGATTCGGATGGTCAGACTAGATGGTGAATCCTGCGGTCGTTCGTACATTGAGGAATACTTAGGTGACTTACGGTCCCTTGAAAATCTCCAAGAGGCTATCGTCAAGATGTca                                                                                                                                                                        >  1:214360/1‑300 (MQ=255)`  
                                                                                                                                            `ggACATCCGTAAGGCTGTAGACGGTCAAGGTGGTGAGAAGAAAGCTGATGAGACAATCGACGTTTACACTCACATCT‑ATCTGGATGAGGACTCAGGTGAATACCT‑CCGATACGAAGAGGTCGAGGGTATGGAAGTCCAAGGCTCCGATGGGACTTATCCTACAGAGGCTTGCCCATACATCCCGATTCGGATGGTCAGACTAGATGGTGAATCCTACGGTCGTTCGTACATTGAGGAATACTTAGGTGACTTACGGTCCCTTGAAAATCTCCAAGAGGCTATCGTCAAGATGTCCATGAtt                                                                                                                                                                  <  1:218370/301‑1 (MQ=255)`  
                                                                                                                                                 `gccGTAAGGCTGTAGAAGGTCAAGGTGGTGAGAAGAAAGCTGATGAGACAATCGACGTGTACACTCACATCT‑ATCTGGATGAGGACTCAGGTGAATACCT‑CCGATACGAAGAGGTCGAGGGTATGGAAGTCAAAGGCTCCGATGGGACTTATCCTAAAGAGGCTTGCCCATACATCCCGATTCGGATGGTCAGACTAGATGGTGAATCCTACGGTCGTTCGTACATTGAGGAATACTTAGGTGACTTACGGTCCCTTGAAAATCTCCAAGAGGCTATCGTCAAGATGTCCATGATTAGctc                                                                                                                                                             <  1:378895/300‑1 (MQ=255)`  
                                                                                                                                                      `atgtgtataagagacagaGGTGGTGAGAAGAAAGCTGATGAGACAATCGACGTGTACACTCACATCT‑ATCTGGATGAGGACTCAGGTGAATACCT‑CCGATACGAAGAGGTCGAGGGTATGGAAGTCCAAGGCTCCGATGGGACTTATCCTAAAGAGGCTTGCCCATACATCCCGATTCGGATGGTCAGACTAGATGGTGAATCCTACGGTCGTTCGTACATTGAGGAATACTTAGGTGACTTACGGTCCCTTGAAAATCTCCAAGAGGCTATCGTCAAGATGTCCATGATTAGCTCTAAgg                                                                                                                                                        <  1:100220/284‑1 (MQ=255)`  
                                                                                                                                                            `gTAGAAGGTCAAGGTGGTGAGAAGAAAGCTGATGAGACAATCTACGTGTACACTCACATCT‑ATCTTGATGAGTACTCAGGTGAATACCT‑CCGATACGAAGAGGTCGAGGGTATGGCAGTCCAAGGCTCCGATGTGACTTATCCTAAAGATGCTTGCCCATACATCCCGCTTCGGATGGTCAGACTAGATGGTGAATCCTACGGTCGTTCGTACATTGAGGAATACTTAGGTGACTTACTGTCCCTTTACAATCTCCAAGAGGCTATCGTCACTATGTCCATGATTATCCCTactgctgtct                                                                                                                                                  >  1:70430/1‑292 (MQ=255)`  
                                                                                                                                                                         `gtggtgAGAAGAAAGCTGATGAGACAATCGACGTGTACACCCCCATCT‑ATCTGGATGAGGACCCAGGTGAATACCT‑CCGATACGAAGAGGTAGAGGGTATGGAAGTCCAAGGCTCAGATGGGACTTATCCTACAGAGACTTGCCCATACATCTCGAATCGGACGGTCAGAATAGATGGTGAATCATACGGTCGTTCGTACATTGAGGAATACTTAGGTGACTTACGGTCACTTGAAAATCTCCAGGGGGCTATCGTCAAGATGTCCATGATTAGCCCTAAGGTTATCGGCTTAGTGAAtac                                                                                                                                     >  1:227714/1‑299 (MQ=255)`  
                                                                                                                                                                            `gtgAGAAGAAAGCTGATGAGACAATCGACGTGTACACTCACATCT‑ATCTGGATGAGGACTCAGGTGAATACCT‑CCGATACGAAGAGGTCGAGGGTATGGAAGTCCAAGGCTCCGATGGGACTTATCCTAAAGAGGCTTGCCCATACATCCCGATTCGGATGGTCAGACTAGATGGTGAATCCTACGGTCGTTCGTACATTGAGGAATACTTAGGTGACTTACGGTCCCTTGAAAATCTCCAAGAGGCTATCGTCAAGCTGTCCATGATTAGCTCTAAGGTTATCGGCTTAGTGAATCctgc                                                                                                                                  >  1:249311/1‑301 (MQ=255)`  
                                                                                                                                                                                `gaagaaAGCTGATGAGACAATCGACGTGTACACTCACATCT‑ATCTGGATGAGGACTCAGGTGAATACCT‑CCGATACGAAGAGGTCGAGGGTATGGAAGTCCAAGGCTCCGATGGGACTTATCCTAAAGAGGCTTGCCCATACATCCCGATTCGGATGGTCAGACTAGATTGTGAATCCTACGGTCGTTCGTACATTGAGGAATACTTAGGTGACTTACGGTCCCTTGAAAATCTCCAAGAGGCTATCGTCAAGATGTCCATGATTAGCTCTACGTTTATCGGCTTCGTGactgtctcttat                                                                                                                              >  1:475009/1‑290 (MQ=255)`  
                                                                                                                                                                                 `aagaaAGCTGATGAGACAATCGACGTGTACACTCACATCT‑ATCTGGATGAGGACTCAGGTGAATACCT‑CCGATACGAAGAGGTCGAGGGTATGGAAGTCCAAGGCTCCGATGGGACTTATCCTAAAGAGGCTTGCCCATACATCCCGATTCGGATGGTCAGACTAGATGGTGAATCCTACGGTCGTTCGTACATTGAGGAATACTTAGGTGACTTACGGTCCCTTGAAAATCTCCAAGAGGCTATCGTCAAGATGTCCATGATTAGCTCTAAGGTTATCGGCTTAGTGAATCCTGCTGGTa                                                                                                                             <  1:194163/301‑1 (MQ=255)`  
                                                                                                                                                                                  `ataaaGCTTATGAGACAATCGACGTGGACACCGACATCT‑ATCTGGATGAGGACTCAGGTGAATACGT‑CCGATACGAAGAGGTCGAGGGTATGGAAGTCCAAGGCTCCGATGGGACTTATCCTAAAGAGGCTTGCCCATACATCCCTATTCGGATGGTCAGACTAGATGGTGAATCCTACGGTCGTTCGTACATTGAGGAATACTTAGGTGACTTACGGTCCCTTGAAAATCTCCAAGAGGCTATCGTCAAGATGTCCATGATTAGCTCTAAGGTTATCGGCTTAGTGAATCCTGCTGGTAt                                                                                                                            <  1:112745/299‑1 (MQ=255)`  
                                                                                                                                                                                             `gagaCAATCGACGTGTACACTCACATCT‑ATCTGGATGAGGACTCAGGTGAATACCT‑CCGATACGAAGAGGTCGAGTGTATGGAAGTCCAAGGCTCCGATGGGACTTATCCTAAAGAGGCTTGCCCATACATCCCGATTCGGATGGTCAGACTAGATGGTGAATCCTACGGTCGTTCGTACATTGAGGAATACTTAGGTTACTTACGGTCCCTTGAAAATCTCCAAGAGGCTATCGTCAAGATGTCCATGATTAGCTCTAAGGTTATCGGCTTAGTGAATCCTGCTGGTATCACCCAGCCAc                                                                                                                 <  1:185880/301‑1 (MQ=255)`  
                                                                                                                                                                                               `aaCAATCGACGTGTACACTCACATCT‑ATCTGGATGAGGACGCAGGTGAATACCT‑CCGATACGAAGAGGTCGAGGGTATGGAAGTCCAAGGCTCCGATGGGACTTATCCTACAGAGGCTTGCCCATACATCCCGATTCGGATGGTCAGACTAGATGGTGAATCCTACGGTCGTTCGTACATTGAGGAATACTTAGGTGACTTACGGTCCCTTGAAAATCTCCAAGAGGCTATCGTCAAGATGTCCATGATTAGCTCTAAGGTTATCGGCTTAGTGAATCCTGCTGGTATCACCCAGCCACGc                                                                                                               <  1:382627/300‑1 (MQ=255)`  
                                                                                                                                                                                                 `cAATCGACGTGTACACTCACATCT‑ATCTGGATGAGGACTCAGGTGAATACCT‑CCGATACGAAGAGGTCGAGGGTATGGAAGTCCAAGGCTCCGATGGGACTTATCCTAAAGAGGCTTGCCCATACATCCCGATTCGGATGGTCAGACTAGATGGTGAATCCTACGGTCGTTCGTACATTGAGGAATACTTAGGTGACTTACGGTCCCTTGAAAATCTCCAAGAGGCTATCGTCAAGATGTCCATGATTAGCTCTAAGGTTATCGGCTTAGTGAATCCTGCTGGTATCACCCAGCCACGCCg                                                                                                             <  1:155091/301‑1 (MQ=255)`  
                                                                                                                                                                                                   `aTCGACGTGTACACTCACATCT‑ATCTGGATGAGGACTCAGGTGAATACCT‑CCGATACGAAGAGGTCGAGGGTATGGAAGTCCAAGGCTCCGATGGGACTTATCCTACAGAGGCTTGCCCATACATCCCGATTCGGATGGTCAGACTAGATGGTGAATCCTACGGTCGTTCGTACATTGAGGAATACTTAGGTGACTTACGGTCCCTTGAAAATCTCCAAGAGGCTATCGTCAAGATGTCCATGATTAGCTCTAAGGTTATCGGCTTAGTGAATCCTGCTGGTATCACCCAGCCACGCCGAc                                                                                                           >  1:91528/1‑301 (MQ=255)`  
                                                                                                                                                                                                   `aTCGACGTGTACACTCACATCT‑ATCTGGATGAGGACTCAGGTGAATACCT‑CCGATACGAAGAGGTCGAGGGTATGGAAGTCCAAGGCTCCGATGGGACTTATCCTAAAGAGGCTTGCCCATACATCCTGATTCGGATGGTCAGACTAGATGGTTAATCCTACGGTCGTTCGTACATTGAGGAATACTTAGGTGACTTACGGTCCCTTGAAAATCTCCAAGAGGCTATCGTCAAGATGTCCATGATTAGCTCTACGGTTATCGGCTTAGTGAATCCTGCTGGTCTCAcccctgtctcttata                                                                                                           >  1:181278/1‑289 (MQ=255)`  
                                                                                                                                                                                                             `acacTAACATCT‑ATCTGGATGAGGATTAAGGTGAATACTT‑CAGATACGAAGAGGTCGAGGGTATGGAAGTCCAAGGCTCGGATGGGACTTATCCTACAGAGGCTTGCCCATACATCCCGATTCGGATGGTCAGACTAGATGGTGAATCCTACGGTCGTTCGTACATTGAGGAATACTTAGGTGACTTACGGTCCCTTGAAAATCTCCAAGAGGCTATCGTCAAGATGTCCATGATTAGCTCTAAGGTTATCGGATTAGTGAATCCTGCTGGTATCACCCAGCCACGCCGACTGACCAAAGc                                                                                                 <  1:489279/301‑1 (MQ=255)`  
                                                                                                                                                                                                              `cacTCACATCT‑ATCTGGATGAGGACTCAGGTGAATACCT‑CCGATACGAAGAGGTCGAGGGTATGGAAGTCAAAGGCTCCGATGGGACTTATCCTAAAGAGGCTTGCCCATACATCCCGATTCGGATGGTCAGACTAGATGGTGAATCCTACGGTCGTTCGTACATTGAGGAATACTTAGGTGACTTACGGTCCCTTGAAAATCTCCAAGAGGCTATCGTCAAGATGTCCATGATTAGCTCTAAGGTTATCGGCTTAGTGAATCCTGCTGGTATCACCCAGCCACGCCGACTGACCAAAGcc                                                                                                >  1:16431/1‑300 (MQ=255)`  
                                                                                                                                                                                                               `acTCACATCT‑ATCTGGATGAGGACTCAGGTGAATACCT‑CCGATACGAAGAGGTCGAGGGTATGGAAGTCCAAGGCTCCGATGGGACTTATCCTAAAGAGGCTTGCCCATACATCACGATTCGGATGGTCAGACTAGATGGTGAATCCTACGGTCGTTCGTACATTGAGGAATACTTAGGTGATTTACGGTCCCTTGAAAATCTCCAAGAGGCTATCGTCAAGATGTCCATGATTAGCTCTAAGGTTATCGGCTTAGTGAATCCTGCTGGTCTCACACAGCCACGCCGACTGCCCAAACCTc                                                                                               >  1:255853/1‑301 (MQ=255)`  
                                                                                                                                                                                                                `cTCACATCT‑ATCTGGATGAGGACTCAGGTGAATACCT‑CCGATACGAAGAGGTCGAGGGTATGGAAGTCCAAGGCTCCGATGGGACTTATCCTAAAGAGGCTTGCCCATACATCCCGATTAGGATGGTCAGACTAGATGGTGAATCCTACGGTAGTTCGTACATTGAGGACTACTTAGGTGACTTACGGTCCCTTGAAAATCTCCAAGAGGCTATCGTCAAGATGTCCATGATTAGCTCTCAGGTTATCGGCTTAGTGACTCCTGCAGGTATCACCAAGCCAAGCCGACTGACAAAAGccag                                                                                              >  1:335312/1‑298 (MQ=255)`  
                                                                                                                                                                                                                    `caTCT‑ATCTGGATGAGGACTCAGGTGAATACCT‑CAGATACGAAGAGGTCGAGGGTATGGAAGTCCAAGGCTCCGATGGGACTTATCCTAAAGAGGCTTGCCCATACATCCCGATTCGGATGGTCAGACTAGATGGTGAATCATACGGTCGTTCGTACATTGAGGAATACTTAGGTGACTTACGGTCCCTTGAAAATCTCCAAGAGGCTATCGTCAAGATGTCCATGATTAGCTCTAAGGTTATCGGCTTAGTGAATCCTGCTGGTATCACCAAGCCACGCCGACTGACCAAAGCTCAGAcc                                                                                          <  1:271827/301‑2 (MQ=255)`  
                                                                                                                                                                                                                      `tct‑atctGGATGGGGACTCAGGTGAATACCT‑CCGATACGAAGAGGTCGAGGGTATGGAAGTCCAAGGCTCCGATTGGACTTATCCTAAAGAGGCTTGCCCATACATCCCGATTCGGATGGTCATACTAGATGGTGAATCCTACGGTCGTTCGTACATTTAGGAATACTTAGGTGACTTACGGTCCCTTGAAAATCTCCAAGAGGCTATCGTCAAGATGTCCATTATTAGCTCTAAGGTTATCGGCTTAGTGAATCCTGCTGGTATCACCCAGCCACGCCGACTGACCAAAGCTCAGACTgg                                                                                        <  1:344014/301‑1 (MQ=255)`  
                                                                                                                                                                                                                       `tcgatctTGATGAGGACGAAGGTGAATACCT‑ACGATACGAAGAGGTCGATAGGAGGGAAGTCAAAGGCTCCGATGGGACTTATCCTAAAGAGGCTGGCCGATTCATCCGGATTCTGATGGTAAGACTAAATGGGGAATCCGACGGTCGTTCGTACATTGAGGAATAGTTAGGTGACTTACGGTCCCTTGAAAATCTCCAAGAGGCTATCGTCAAGATGTCCATGATTAGCTCTAAGGTTATCGGCTGAGTGAATCCTGCTGGTATCACCCAGCCACTCCGACTGACCAAAGCTCAGACTgg                                                                                        <  1:75087/298‑1 (MQ=255)`  
                                                                                                                                                                                                                       `ct‑atctGGATGAGGACTCAGGTGAATACCT‑CCGATACGAAGAGGTCGAGGGTATGGAAGTCCAAGGCTCCGATGGGACTTATCCTAAAGAGGCTTGCCCATACATCCCGATTCGGATGGTCAGACTAGATGGTGAATCCTACGGTCGTTCGTACATTGAGGAATACTTAGGTGACTTACGGTCCCTTGAAAATCTCCAAGAGGCTATCGTCAAGATGTCCATGATTAGCTCTAAGGTTATCGGCTTAGTGAATCCTGCTGGTATCACCAAGCCACGCCGACTGACCAAAGcctgtctctta                                                                                       >  1:455391/1‑291 (MQ=255)`  
                                                                                                                                                                                                                        `t‑atctGGATGAGGACTAAGGTGAATACCT‑CCGATACGAAGAGGTCGAGGGTATGGAAGTCCAAGGCTCCGATGGGAATTATCCTAAAGAGGCTTGCCCATACATCCCGATTCGGATGGTCAGACTAGATGGTGAATCCTACGGTCGTTCGTACATTGAGGAATACTTAGGTGACTTACGGTCCCTTGAAAATCTCCAAGAGGCTATCGTCAAGATGTCCATGATTAGCTCTAAGGTTATCGGCTTAGTGAATCCTGCTGGTATCACCCAGCCACGCCGACTGACCAAAGCTCAGACTGGTg                                                                                      <  1:408967/301‑1 (MQ=255)`  
                                                                                                                                                                                                                          `atctGGATGAGGACTCAGGTGAATACCT‑CCGATACGAAGAGGTCGAGGGTATGGAAGTCCAAGGCTCCGATGGGACTTATCCTACAGAGGCTTGCCCATACATCCCGATTCGGATGGTCAGACTAGATGGTGAATCCTACGGTCGTTCGTACATTGAGGAATACTTAGGTGACTTACGGTCCCTTGAAAATCTCCAAGAGGCTATCATCAAGATGTCCATGATTAGCTCTAAGGTTATCGGCTTAGTGAATCCTGCTGGTATCACCCAGCCACGCAGACTGACCAAAGCTCAGACTGGTgg                                                                                     >  1:265675/1‑300 (MQ=255)`  
                                                                                                                                                                                                                           `tctGGATGAGGACTCAGGTGAATACCT‑CCGATACGAAGAGGTCGAGGGTATGGAAGTCCAAGGCTCCTATGGGACTTATCCTACAGAGGCTTGCTCATACATCCCGATTCGGATGGTCAGACTAGATGGTGAATCCTACGGTCGTTCGTACATTGAGGAATACGTAGGTGACTTACGGTCCCTTGAAAATCTCCAAGAGGCTATCGTCAAGATGTCCATGATTAGCTCTAAGGTTATCGGCTTAGTGAATCCTGCTGGTATCACCCAGCCACGCCGACTTACCAAAGCTCAGACTGGTGAc                                                                                    <  1:232492/301‑1 (MQ=255)`  
                                                                                                                                                                                                                               `tatcagggacaggggtaaTACCT‑CCGATACGAAGAGGTCGAGGGTATGGAAGTCCAAGGCTCCGATGGGACTTATCCTAAAGAGGCTTGCCCATACTTCCCGATTCGGATGGTCAGACTAGATGGTGAATCCTACGGTCGTTCGTACATTGAGGAATACTTAGGTGACTTACGGTCCCTTGAAAATCTCCAAGAGGCTATCGTCAAGATGTCCATGATTAGCTCTAAGGTTATCGGCTTAGTGAATCCTGCTGGTATCACCCAGCCACGCCGACTGACCAAAGCTCAGACTGGTGACTTCg                                                                                <  1:27708/285‑1 (MQ=255)`  
                                                                                                                                                                                                                                   `gggACTCAGGTGAATACCT‑CCGATACGAAGAGGTCGAGGGTATGGAAGTCCAAGGCTCCGATGGGACTTATCCTAAAGAGGCTTGCCCATACATCCCGATTCGGATGGTCAGACTAGATGGTGAATCCTACGGTCGTTCGTACATTGAGGAATACTTAGGTGACTTACGGTCCCTTGAAAATCTCCAAGAGGCTATCGTCAAGATGTCCATGATTAGCTCTAAGGTTATCGGCTTAGTGAATCCTGCTGGTATCACCCAGCCACGCCGACTGACCAAAGCTCAGACTGGTGACTTCGTTAc                                                                            <  1:205072/300‑1 (MQ=255)`  
                                                                                                                                                                                                                                   `aGGACTCAGGTGAATACCT‑CCGATACGAAGAGGTCGAGGGCATGGAAGTCCAAGGCCCCGATGGCACTCATCCTAAAGACGCTTGCCCATACAACCCGATTCGGATGGTCAGACTAGATGGTGAATCATACGTTCGTTCGTTCATTGAGGAATACTTAGATGACTTACGGTCCTTTGAAACTCTCCACGCGGCTATCGTCAAGATGTCCATGATTAGCTCTAAGGTTATCGGCTTAGTGAATCCTGCTGGTATTACCCAACCACCCCGACTGACCAAAGCTCAGCCTGGTAACTTCGTTat                                                                            >  1:3111/1‑300 (MQ=255)`  
                                                                                                                                                                                                                                       `gagactggcattccT‑CCGATACGAAGAGGTCGAGGGTATGGAAGTCCAAGGCTCCGATGGGACTTATCCTAAAGAGGCTTGCCCATACATCCCGATTCGGATGGTCAGACTAGATGGTGAATCCTACGGTCGTTCGTACATTGAGGAATACTTAGGTGACTTACGGTCCCTTGAAAATCTCCAAGAGGCTATCGTCAAGATGTCCATGATTAGCTCTAAGGTTATCGGCTTAGTGAATCCTGCTGGTATCACCCAGCCACGCCGACTGACCAAAGCTCAGACTGGTGACTTCGTTACTGgt                                                                        <  1:209747/289‑1 (MQ=255)`  
                                                                                                                                                                                                                                       `acagggTGAATACCT‑CAGATACGAAGAGGTCGAGGGTATGGAAGTCCAAGGCTCCGATGGGACTTATCCTAAAGAGGCTTGCCCATACATCCCGATTCGGATGGTCAGACTAGATGGTGAATCCTACGGTCGTTCGTACATTGAGGAATACTTAGGTGACTTACGGGCCCTTGAAAATCTCCAAGAGGCTATCGTCAAGATGTCCATGATTAGCTCTAAGGTTATCGGCTTAGTGAATCCTGCTGGTATCACCCAGCCACGCCGACTGACCAAAGCTCAGACTGGTGACTTCGTTACTGgt                                                                        <  1:413040/297‑1 (MQ=255)`  
                                                                                                                                                                                                                                           `ggTGAATACCT‑CCGATACGAAGAGGTCGAGGGTATGGAAGTCCAAGGCTCCGATGGGACTTATCCTAAAGAGGCTTGCCCATACATCCCGATTCGGATGGTCAGACTAGATGGTTAATCCTACGGTCGTTCGTACATTGAGGAATACTTAGGTGAATTACGGTACCTTGAAAATCTCCAAGAGGCTATCGTCAAGATGTACATGATTAGCTCTAAGGTTATCGGCTTAGTGAATCCTTCTGGTATAACCCAGCCACGCCAGCTGACCAAAGCTCAGACTGGTGACATCGTTACTGgtcgtc                                                                    >  1:302258/1‑301 (MQ=255)`  
                                                                                                                                                                                                                                           `ggTGAATACCT‑CCGATACGAAGAGGTCGAGGGTATGGAAGTCCAAGGCTCCGATGGGACTTATCCTAAAGAGGCTTGCCCATACATCCCGATTCGGATGGTCAGACTAGATGGTGAATCCTACGGTCGTTCGTACATTGAGGAATACTTAGGTGACTTACGGTCCCTTGAAAATCTCCAAGAGGCTATCGTCAAGATGTCCATGATTAGCTCTAAGGTTATTGGCTTAGGGAATCCTGCT‑GTATCACCCAGTCACGCCGACTGACCAAAGCTCAGACTGGTGACTTCGTTACTGGTAGTcc                                                                   >  1:464060/1‑301 (MQ=255)`  
                                                                                                                                                                                                                                            `ttGAATACCT‑CCGATACGAAGAGGTCGAGGGTATGGAAGTCCAAGGCTCCGATGGGACTTATCCTAAAGAGGCTTGCCCATACATCCCGATTCGGATGGTCAGACTAGATGGTGAATCCTACGGTCGTTCGTACATTGAGGAATACTTAGGTGACTTACGGTCCCTTGAAAATCTCCAAGAGGCTATCGTCAAGATGTCCATGATTAGCTCTAAGGTTATCGGCTTAGTGAATCCTGCTGGTATCACCCAGCCACGCCGACTGACCAAAGCTCAGACTGGTGACTTCGTTACTGGTCGTcc                                                                   <  1:450859/300‑1 (MQ=255)`  
                                                                                                                                                                                                                                              `gAATACCT‑CCGATACGAAGAGGTCGAGGGTATGGAAGTCCAAGGCTCCGATGGGACTTATCCTAAAGAGGCTTGCCCATACATCCCGATTCGGATGGTCAGACTAGATGGTGAATCCTACGGTCGTTCGTACATTGAGGAATACTTAGGTGACTTACGGTCCCTTGAAAATCTCCAAGAGGCTATCGTCAAGATGTCCATGATTAGCTCTAAGGTTATCGGCTTAGTGAATCCTGCTTGTATCACCCAGCCACGCCGACTGACCAAAGCTCAGACTGGTGACTTCGTTACTGGTCGTCCag                                                                 >  1:123950/1‑301 (MQ=255)`  
                                                                                                                                                                                                                                                              `gAAGAGGTCGAGGGTATGGAAGTCCAAGGCTCCGATGGGACTTATCCTAAAGAGGCTTGCCCATACATCCCGATTCGGATGGTCAGACTAGATGGTGAATCCTACGGTCGTTCGTACATTGAGGAATACTTAGGTGACTTACGGTCCCTTGAAAATCTCCAAGAGGCTATCGTCACGATGTCCATGATTAGCTCTAAGTTTATCGGCTTAGTGAATCCTTCTGGTAGCACCCAGCAACGCCGACTGACCAAAGCTCAGACTGGTGACTTCGTTACTGGTCGTCCAGAAGAcctgtcccttt                                                  >  1:495900/1‑291 (MQ=255)`  
                                                                                                                                                                                                                                                                   `ggTCGAGGGTATGGAAGTCCAAGGCTCCGATGGGACTTATCCTAAAGAGGCTTGCCCATACATCCCGATTCGGATGGTCAGACTAGATGGTGAATCCTACGGTCGTTCGTACATTGAGGAATACTTAGGTGACTTACGGTCCCTTGAAAATCTCCAAGAGGCTATCGTCAAGATGTCCATGATTAGATCTAAGGTTATCGGCTTAGTGAATCCTACTGGTATCACCCAGCCACGCCGACTGACCAAAGCTCAGACTGGTGACTTCGTTACTGGTCGTCCAGAAGACATCTCGGtcctgtct                                             >  1:104522/1‑297 (MQ=255)`  
                                                                                                                                                                                                                                                                      `cGAGGGTATGGTAGCCCAAGGTCCCGTTGGGCCTTATCCTAAAGAGGCTTGCCCATACATCCCGATTCGGGTGGTCAGACTAGATGGTGAATCCTACGGTCGTTCGTACATTGAGGAATACTTAGTTGAATTACGGTCCCTTGAAAATCTCCAAGAGGCTATCGTCAAGATGTCCATGATTAGCTCTAAGGTTATCGGCTTAGTGAATCCTGCTGGTATCACCCAGCCACGCCGACTGACCAAAGCTCAGACTGGTGACTTCGTTACTGGTCGTCCAGAAGACATCTCGTTCCTCCAACTg                                          <  1:432968/301‑1 (MQ=255)`  
                                                                                                                                                                                                                                                                                 `aaGTCCAAGGCTCCGATGGGACTTATCCTAAAGAGGCTTGCCCATACATCCCGATTCGGATGGTCAGACTAGATGGTGAATCCTACGGTCGTTCGTACATTGAGGAATACTTAGGTGACTTACGGTCCCTGGAAAATCTCCAAGAGGCTATCGTCAAGATGTCCATGATTAGCTCTAAGGTTATCGGCTTAGTGAATCCTGCTGGTATCACCCAGCCACGCCGACTGACCAAAGCTCAGACTGGTGACTTCGTTACTGGTCGTCCAGAAGACATCTCGTTCCTCCAACTGGAGaagaaagc                               <  1:66689/301‑1 (MQ=255)`  
                                                                                                                                                                                                                                                                                          `gCTCCGATGGGACTTATCCTAAAGAGGCTTGCCCATACATCCCGATTCGGATGGTCAGACTAGATGGTGAATCCTACGGTCGTTCGTACATTGAGGAATACTTAGGTGACTTACGGTCCCTTGAAAATCTCCAAGAGGCTATCGTCAAGATGTCCATGATTAGCTCTAAGGTTATCGGCTTAGTGAATCCTGCTGGTATCACCCAGCCACGCCGACTGACCAAAGCTCAGACTGGTGACTTCGTTACTGGTCGTCCAGAAGACATCTCGTTCCTCCAACTGGAGAAGCCAGCAGACTTTAc                      >  1:171490/1‑301 (MQ=255)`  
                                                                                                                                                                                                                                                                                            `cccGATTGGACTTATCCTAAAGAGGCTTGCCCATACATCCCGATTCGGATGGTCAGACTAGATGGTGAATCCTACGGTCGTTCGTACATTGAGGAATACTTAGGTGCCTTACGGTCCCTTGAAAATCTCCAAGAGGCTATCGTCAAGATGTCCATGATTAGCTCTAAGGTTATCGGCTTAGTGAATCCTGCTGGTATCACCCAGCCACGCCGACTGACCAAAGCTCAGACTGGTGACTTCGTTACTGGTCGTCCAGAAGACATCTCGTTCCTCCAACTGGAGAAGCAAGCAGACTTTACTg                    <  1:326991/300‑1 (MQ=255)`  
                                                                                                                                                                                                                                                                                            `cccGATGGGACTTATCCTAAAGAGGCTTGCCCATACATCCCGATTCGGATGGTCAGACTAGATGGTGAATCCTACGGTCGTTCGTACATTGAGGAATACTTAGGTGACTTACGGTCCCTTGAAAATCTCCAAGAGGCTATCGTCAAGATGTCCATTATTAGCTCTAAGGTTATCGGCTTAGTGAATCCTGCTGGTATCACCCAGCCACGCCGACTGACCAAAGCTCAGACTGGTGACTTCGTTACTGGTCGTCCAGAAGACATCTCGTTCCTCCAACTGGAGAAGCAAGCAGACTTTACTg                    <  1:387684/300‑1 (MQ=255)`  
                                                                                                                                                                                                                                                                                             `ccGATGGGACTTATCCTAAAGAGGCTTGCCCATACATCCAGATTCGGATGGGCAGACTAGATGGTGAATCCTACGGTCGTTCGTACATTGAGGAATACTTAGGTGACTTACGGTCCCTTGAAAATCTCCAAGAGGCTATCGTCAAGATGTCCATGATTAGCTCTAAGGTTATCGGCTTAGTGAATCCTGCTGGTATCACCCAGCCACGCCGACTGACCAAAGCTCAGACTGGTGACTTCGTTACTGGTCGTCCAGAAGACATCTCGTTCCTCCAACTGGAGAAGCAAGCAGACTTTACTGt                   >  1:320591/1‑301 (MQ=255)`  
                                                                                                                                                                                                                                                                                                 `tGGGTCTTATCCTACAGAGGCTTGCCCATACATCCCGAATCGGATGGTCAGACTAGATGGTGAATCCTACGGTAGTTCGTACATTGAGGAATACTTAGGTGACTTACGGTCTCGTGAAAATCTCCAAGAGGCTATCGTCAAGATGCCCATGATTAGCTCTAAGGTTATCGGCTTAGTGAATCCTGCTGGTATCACCCAGCCACGCCGACTGACCAAATCTCAGACTGGTGACTTCGTTACTGGTCGTCCAGAAGACATCTCGTTCCTCCAACTGGAGAAGCAAGCAGACTTTACTGTAGCt               <  1:459570/301‑1 (MQ=255)`  
                                                                                                                                                                                                                                                                                                 `agagacagaTCCTAAAGAGGCTTGCCCATACATCCCGATTCGGATGGTCAGACTAGATGGTTAATCCTACGGTCGTTCGTACATTGAGGAATACTTAGGTGACTTACGGTCCCTTGAAAATCTCCAAGAGGCTATCGTCAAGATGTCCATGATTAGCTCTAAGGTTATCGGCTTAGTGAATCCTGCTGGTATCACCCAGCCACTCCTACTGACCAAAGCTCAGACTGGTGACTTCGTTACTGGTCGTCCAGAAGACATCTCGTTCCTCCAACTGGAGAAGCAAGCAGACTTTACTGTAGCt               <  1:361664/293‑1 (MQ=255)`  
                                                                                                                                                                                                                                                                                                      `cTTATCCTAAAGAGGCTTGCCCATACATCCCGATTCGGATGGTCAGACTAGATGGTGAATCCTACGGTCGTTCGTACATTGAGGAATACTTAGGTGACTTACGGTCCCTTGAAAATCTCCAAGAGGCTATCGTCAAGATGTCCATGATTAGCTCTAAGGTTATCGGCTTAGTGAATCCTGCTGGTATCACCCAGCCACGCCGACTGACCAAAGCTCAGACTGGTGACTTCGTTACTGGTCGTCCAGAAGACATCTCGTTCCTCCAACTGGAGAAGCAAGCCGACTTTACTGTaaccatgtc          >  1:282559/1‑293 (MQ=255)`  
                                                                                                                                                                                                                                                                                                         `aTCCTAAAGAGGCTTGCCCATACATCCCGATTCGGATGGTCAGACTAGATGGTGAATCCTACGGTCGTTCGTACATTGAGGAATACTTAGGTGACTTACGGTCCCTTGAAAATCTCCAAGTGGCTATCGTCAAGTTGTCCATGATTAGCTCTAAGGTTATCGGCTTAGTGAATCCTGCTTGTATCACCCATCCACGCCGACTTACCAAATCTCAGACTGTTGACTTCGTTACTGTTCGTCCAGAAGACATCTCGTTCCTCCTCCTGGAGCATCAAGCAGACTTTACTTTATCTATAGcctt       >  1:417042/1‑299 (MQ=255)`  
                                                                                                                                                                                                                                                                                                          `tCCTAAAGAGGCTTGCCCATACATCCCGATTCGGATGGTCAGACTAGATGGTGAATCCTACGGTCGTTCGTACATTGAGGAATACTTAGGTGACTCACGGTCCCTTGAAAATCTCCAAGAGGCTATCGTCAAGATGTCCATGATTAGCTCTAATGTTGTCGGCTTAGTGAATCCTGCTGGTATCACCCAGCCACGCCGACTGACCAAAGCTCAGACTGGTGACTTCGTTACTGGTCGTCCAGAAGACATCTCGTTCCTCCAACTGGAGAAGCAACCAGACGTTACTGTAGCCAAAGCTGtc      >  1:31657/1‑300 (MQ=255)`  
                                                                                                                                                                                                                                                                                                           `ccTAATGAGTCTTGCCCATACATCCCGATTCTGATGGTCAGACTAGATGGTGAATCCTACGGTCGTTCGTACATTGAGGAATACTTAGGTGACTTACGGTCCCTTGAAAATCTCCAAGAGGCTATCGTCAAGATGTCCATGATTAGCTCTAAGGTTATCGGC‑TAGTGAATCCTGCTGGTATCACCCAGCCACGCCGACTGACCAAAGCTCAGACTGGTGACTTCGTTACTGGTCGTCCAGAAGACATCTCGTTCCTCCAACTGGAGAAGCAAGCAGACTTTACTGTAGCTAAAGCCGTAAg    <  1:359/301‑1 (MQ=255)`  

                                                                                                                                                                                                                                                                                                               `|`                                                                                                                                                                                                                                                                                                             
`ACCTACCGGAACCGGAAGGGTCAAACTATAATCCCATGAAGCTGTACCGATTGTCTTCTTATGTGGTCCAACGAGACGCATTCGGCAACGTTCTGCAAATGGTGACTCGTGACCAGATAGCTTTTGGTGCTCTCCC‑TGAGGACATCCGTAAGGCTGTAGAAGGTCAAGGTGGTGAGAAGAAAGCTGATGAGACAATCGACGTGTACACTCACATCT‑ATCTGGATGAGGACTCAGGTGAATACCT‑CCGATACGAAGAGGTCGAGGGTATGGAAGTCCAAGGCTCCGATGGGACTTATCCTAAAGAGGCTTGCCCATACATCCCGATTCGGATGGTCAGACTAGATGGTGAATCCTACGGTCGTTCGTACATTGAGGAATACTTAGGTGACTTACGGTCCCTTGAAAATCTCCAAGAGGCTATCGTCAAGATGTCCATGATTAGCTCTAAGGTTATCGGCTTAGTGAATCCTGCTGGTATCACCCAGCCACGCCGACTGACCAAAGCTCAGACTGGTGACTTCGTTACTGGTCGTCCAGAAGACATCTCGTTCCTCCAACTGGAGAAGCAAGCAGACTTTACTGTAGCTAAAGCCGTAAGTG  >  NC_001604.1/20676‑21275`

  

Alignment Legend

Aligned base mismatch/match (shaded by quality score): `ATCG/ATCG` `< 3 ≤` `ATCG/ATCG` `< 9 ≤` `ATCG/ATCG` `< 17 ≤` `ATCG/ATCG` `< 32 ≤` `ATCG/ATCG` `< 38 ≤` `ATCG/ATCG`

Unaligned base: `atcg`    Masked matching base: `atcg`    Alignment gap: `‑`    Deleted base: `‑`
